# High-Frequency Ultrasound of Multiple Arterial Areas Reveals Increased Intima Media Thickness, Vessel Wall Appearance, and Atherosclerotic Plaques in Systemic Lupus Erythematosus

**DOI:** 10.3389/fmed.2020.581336

**Published:** 2020-10-09

**Authors:** Christina Svensson, Per Eriksson, Helene Zachrisson, Christopher Sjöwall

**Affiliations:** ^1^Department of Clinical Physiology, University Hospital, Linköping, Sweden; ^2^Department of Health, Medicine and Caring Sciences, Linköping University, Linköping, Sweden; ^3^Division of Inflammation and Infection, Department of Biomedical and Clinical Sciences, Linköping University, Linköping, Sweden

**Keywords:** systemic lupus erythematosus, ultrasound, IMT, plaque, vessel wall

## Abstract

**Introduction:** Despite improved therapies and management, patients with systemic lupus erythematosus (SLE) still have increased risks of cerebrovascular and cardiovascular disease. High-frequency ultrasound (US) provides an opportunity to distinguish atherosclerosis from inflammation in the vessels. We hypothesized that an extended US protocol may add information regarding vascular affection in SLE.

**Methods:** Sixty patients (52 women, 8 men; mean age 43.2 ± 11.3 years) with SLE characterized by either lupus nephritis (LN; *n* = 20), antiphospholipid syndrome (APS; *n* = 20), or skin and joint involvement (*n* = 20) as well as matched healthy controls (*n* = 60) were included. Intima-media thickness (IMT), assessment of vessel walls, and plaque occurrence were recorded using high-frequency US (GE Logic E9) in common carotid, internal carotid, brachiocephalic, subclavian, axillary, common femoral, and proximal superficial femoral arteries as well as in the aortic arch.

**Results:** For the entire SLE group, IMT was increased in the internal carotid artery (0.52 ± 0.17 vs. 0.45 ± 0.09 mm, *p* = 0.004), the common femoral artery (0.57 ± 0.23 vs. 0.49 ± 0.11 mm, *p* < 0.01), the subclavian artery (0.58 ± 0.19 vs. 0.53 ± 0.13 mm, *p* = 0.02), and the aortic arch (1.21 ± 0.63 vs. 0.98 ± 0.25 mm, *p* = 0.002) compared to controls. These differences were primarily observed in the APS and LN groups compared to controls. Vessels with increased IMT ≥0.9 mm had a smooth, medium echogenic appearance in areas free of atherosclerotic plaques. Atherosclerotic plaques were detected in 15/60 patients (25%) as compared to 2/60 of the controls (3%). Plaques were predominantly (67%) located in the carotid bifurcation. Multivariate analysis revealed influence of age on IMT in all vessel areas. Furthermore, in the common femoral artery, sagittal abdominal diameter, diastolic blood pressure, and cholesterol all showed association with increased IMT. In the internal carotid artery, male sex and presence of Raynaud phenomenon influenced IMT.

**Conclusion:** Among SLE patients without presence of plaques, an extended US protocol revealed increased wall thickness with predominantly medium echogenic appearance highlighting possibly inflammation or early atherosclerosis. The appearance of vessel walls has not previously been studied in detail. An increased number of plaques were found in SLE compared to age- and sex-matched healthy controls. We found similar risk factors for increased IMT and occurrence of plaques, possibly indicating atherosclerotic mechanisms rather than inflammation.

## Introduction

Systemic lupus erythematosus (SLE) is an autoimmune inflammatory disease affecting multiple organ systems which often affects young females (1). Increased morbidity and mortality, particularly from cardiovascular disease (CVD), remains a reality in SLE (2, 3). The increased risk has been estimated to 2- to 10-fold compared to the general population, but as high as 50-fold among female SLE patients aged 35–44 years (4–6). In Sweden, the relative risk of ischemic stroke in SLE is more than doubled compared to the general population (7). According to Gustafsson et al., patients with lupus nephritis (LN) display carotid plaques twice as often as patients without renal involvement and matched controls (3).

Antiphospholipid syndrome antibodies (aPL) form a heterogeneous group of antibodies targeting phospholipid-binding proteins and phospholipids. The aPL included in the APS classification criteria are lupus anticoagulant, anticardiolipin [immunoglobulin G (IgG)/IgM], and anti–β2-glycoprotein I antibodies (IgG/IgM) (8). These antibodies have important roles, e.g., by interfering with the coagulation system. Up to 40% of all SLE cases display elevated levels of any aPL at some point during the course of the disease, yet only approximately half of these SLE cases will fulfill the APS classification criteria over time. Presence of aPL among patients with SLE is associated with acquired organ damage and a more severe course of disease (9, 10).

High-frequency ultrasound (US) provides an opportunity to distinguish atherosclerosis from inflammation in the vessel walls. Measurement of intima-media thickness (IMT) with high-frequency US in the common carotid artery constitutes a validated method to assess early atherosclerosis (11). Different appearance of the vessel walls is seen depending on the cause of vascular affection.

A hypertensive hypertrophic response of medial cells can be seen in early atherosclerosis and can be quantified by IMT-measurement, while plaques are often observed in a later phase and may be related to inflammation, oxidation, endothelial dysfunction and/or smooth muscle cell proliferation (12, 13). Calcification of the intima is a marker of atherosclerotic disease and associates with arterial stenosis, whereas calcification in the media is associated with arterial stiffness in the elastic layer of the medial wall, which can often be seen in diabetes and chronic renal failure (14). High echogenic, irregular IMT implies a more fibrous, calcified vessel wall which is found in atherosclerotic plaques (15). Prominent thickened, circumferential medium echogenic IMT implies early inflammatory vessel wall changes. Wall thickening with or without hyperechogenic stripes lining the innermost wall layer has been regarded as typical signs of arteritis after the acute stage of the disease (16–18). In comparison with other imaging techniques, US has a higher resolution and is thus valuable in the assessment of vessel walls albeit it is operator dependent (19).

Herein, we aimed to evaluate whether an extended US protocol (including examination of carotid arteries, central neck arteries as well as femoral arteries) may add valuable information regarding the type of vessel affection among patients with SLE in a quiescent phase of their disease.

## Materials and Methods

### Subjects

In this cross-sectional study, we included 60 patients (52 women, 8 men; mean age 43.2 ± 11.3 years), diagnosed with SLE based on fulfillment of the 1982 American College of Rheumatology (ACR) and/or the 2012 Systemic Lupus Collaborating Clinics (SLICC) classification criteria as detailed in Supplementary Table 1 (20, 21). Patients above 63 years of age were excluded due to a higher background risk of atherosclerosis dependent on age (22), whereas patients below the age of 23 years were excluded due to an overall short duration of SLE. All patients were followed longitudinally within the frame of an observational research program KLURING (a Swedish acronym for *Clinical LUpus Register In North-eastern Gothia*) at the Rheumatology Unit, Linköping University hospital, as previously detailed (23). Acquired organ damage was assessed by the SLICC/ACR damage index (SDI) and disease activity by the SLE disease activity index 2000 (SLEDAI-2K) for each patient, which was recorded from their closest regular visit to rheumatologist (24, 25). Mean time between examination and disease activity assessment was 3.8 months; 29/60 (48%) patients had a serologically active, but clinically quiescent SLE (26), and 50/60 cases (83%) had Caucasian ancestry.

Sixty patients included in KLURING were selected and divided into 3 phenotypic subgroups with different SLE manifestations. The subgroups were matched between each other 1:1:1 according to sex and age; 20 cases meeting the renal disorder ACR criterion for LN (20) in the absence of APS, 20 cases meeting APS criteria (8) in the absence of LN, and 20 cases with skin and joint involvement in the absence of LN and APS.

Sixty healthy age- and sex-matched (1:1 to the 60 SLE cases), non-medicated controls without clinical signs of inflammatory or atherosclerotic disease (52 women and 8 men; mean age 42.9 ± 11.5 years), were examined using the same protocol as for the patients. The healthy controls were all of Caucasian ethnicity and had all been recruited from the hospital staff.

### Background Variables

We obtained demographic data from all subjects regarding height, weight, waist circumference and sagittal abdominal diameter. Variables concerning age, sex, smoking habits and ongoing pharmacotherapy were collected. Blood pressure was determined with oscillometric technique (Dinamap PRO 200 Monitor, Critikon, Tampa, FL, USA).

### Laboratory Measurements

Standard cardiovascular and inflammatory laboratory test with measurements of total cholesterol, triglycerides, high-density lipoprotein (HDL), low-density lipoprotein (LDL), plasma creatinine and C-reactive protein with high sensitive technique (hsCRP) were collected after 12-h overnight fasting. Presence of serological disease activity, with anti–double-stranded DNA antibodies using addressable laser bead immunoassay (FIDIS™ Connective profile, Solonium software version 1.7.1.0, Theradiag, Croissy-Beaubourg, France) and plasma analyses complement protein 3 (C3) and 4 (C4), was controlled for at the closest regular visit to rheumatologist (27).

#### US

**Figure 1:**
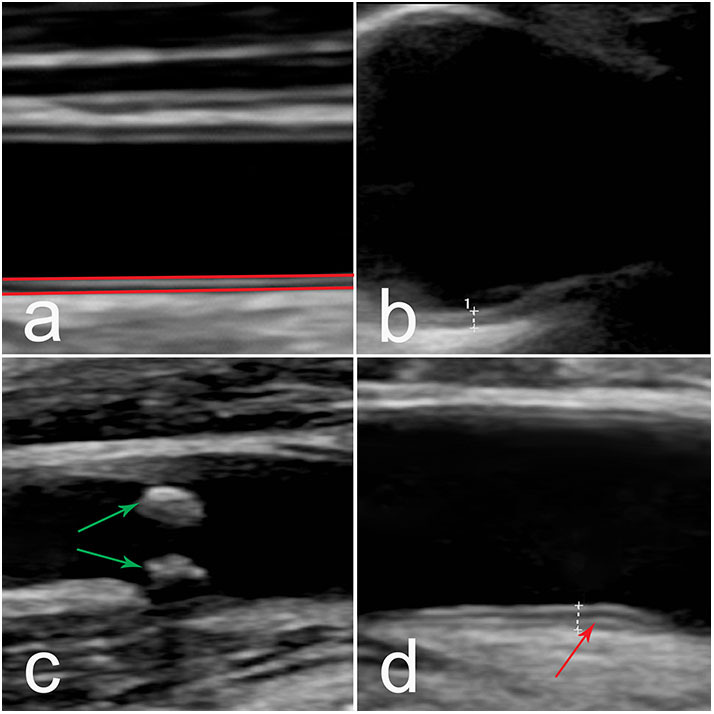
**(a)** Ultrasound image of the CCA. Principle of IMT measurements (red lines) in the vessel. **(b–d)** Ultrasound images of different vessel wall appearance, **(b)** smooth medium echogenic, homogenous wall thickening in the aortic arch, **(c)** atherosclerotic plaque (green arrow) in CCA bifurcation, **(d)** medium echogenic, homogenous wall thickening with fibrotic stripes (red arrow) in CFA. IMT, intima-media thickness; CCA, common carotid artery; CFA, common femoral artery.

For the US measurements, a GE Logic E9 US system (LOGIQ E9 XDclear 2.0 General Electric Medical Systems US, Wauwatosa, WI, USA) with linear transducer L2-9 MHz was used. For the aortic arch, a C1-6 MHz transducer was used. IMT was measured in common carotid artery (CCA), internal carotid artery (ICA), subclavian artery (SCA), axillar artery (AxA), common femoral artery (CFA), superficial femoral artery (SFA) and the aortic arch. Measuring principles are shown in Figure 1a. Both sides were investigated. The procedure has been described previously (28), with an addition of CFA and SFA in this study. For IMT measurements in CCA a 10 mm wide box was placed over the common carotid artery far wall, near (10 mm) the carotid bifurcation. A mean value of all measured far wall points in the box was presented. For validation of the method two repeated measurements were performed. Maximum systolic flow velocity was measured in all vessels to evaluate possible arterial stenosis. Plaques were defined as focal areas in the vessel wall where IMT showed increase of either 0.5 mm or 50% compared to the IMT in the adjacent wall.

In areas free of plaques with IMT ≥0.9 mm for carotid and central arteries, and ≥1.2 mm for the aortic arch, the vessel wall was assessed regarding echogenicity (low–medium–high). Furthermore, distribution and presence of fibrotic stripes were noted. The cutoff value of ≥0.9 mm was chosen due to the latest European Society of Hypertension/European Society of Cardiology (ESH/ESC) hypertension guidelines (29). For the aortic arch a higher cutoff value was chosen due to generally higher aortic arch IMT values among our healthy controls, according to results from earlier studies (30). Plaques were assessed regarding echogenicity (low–medium–high), distribution, irregularity (homogenicity or heterogenicity) and cap (smooth surface or ulceration).

A standardized examination procedure was used in all individuals. The participant had to rest 15 min before the test which was performed in a room with a temperature of 25°C, dim lighting and no outer disturbances. All participants were asked to refrain from coffee 4 h prior to the measurements.

The same vascular sonographer performed all US examinations and offline measurements performed after the exam. The sonographer was blinded to which classification criteria the patients with SLE fulfilled, but not blinded to whether the participants were patients or controls.

### Ethics Considerations

Oral and written informed consent was obtained from all patients and healthy controls. The study protocol was performed according to the Declaration of Helsinki and approved by the Regional Ethics Board in Linköping (ref. M75-08, 2013/33-31 and ref. 2017/572-32).

### Statistical Methods

According to the central limit theorem, sufficiently many subjects were included to allow use of methods that relay on the normal distribution. Demographic values and IMT are presented as mean ± SD. Differences between the whole SLE group and controls were calculated using Student's *t*-test. Differences between subgroups were calculated using one-way ANOVA. For binary variables chi square test was used. Univariate linear regression was used to establish relationship between IMT values and each of the variables in Table 1. Multivariate linear regression and logistic regression were used to examine factors explaining IMT and presence of plaques. All variables significant in the univariate model were combined and a stepwise procedure eliminating non-significant (*p* ≥ 0.05) variables at each step until a multiple model with only significant variables remained was performed. For missing data, no imputation analysis was performed. Statistical analyses were performed using SPSS version 25.0 (IBM, Armonk, NY USA).

**Table 1:** Detailed characteristics of included patients and controls presented as mean±SD or *n* (%).

	**All SLE (*n* = 60)**	**Controls (*n* = 60)**	**LN (*n* = 20)**	**APS (*n* = 20)**	**Skin and joint (*n* = 20)**
	**mean ± SD**	**mean ± SD**	**mean ± SD**	**mean ± SD**	**mean ± SD**
**Background variables**					
Age at examination (years)	43.2 ± 11.3	43.0 ± 11.4	41.6 ± 10.4	45.2 ± 12.2	42.9 ± 11.7
Female gender, *n* (%)	52 (87)	52 (87)	18 (90)	15 (75)	19 (95)
Duration of SLE (years)	12.0 ± 9.4	N/A	10.7 ± 8.1	15.6 ± 12.2	9.6 ± 6.3
SDI	0.8 ± 1.1	N/A	0.6 ± 0.9	1.5 ± 1.4	0.4 ± 0.5
SLEDAI-2K	2.0 ± 2.1	N/A	1.6 ± 2.1	2.1 ± 2.4	2.2 ± 1.7
Serologically active clinically quiescent SLE, *n* (%)	29 (48)	N/A	13 (65)	10 (50)	6 (30)
**Traditional risk factors and laboratory data**					
Body mass index (BMI) (kg/m^2^)	26.0 ± 4.2**	24.0 ± 3.3	26.5 ± 3.4*	25.6 ± 4.0	25.8 ± 5.1
Waist circumference (cm)	92.4 ± 12.1***	83.0 ± 10.0	93.2 ± 11.2**	92.2 ± 12.8*	91.8 ± 12.7
Sagittal abdominal diameter (cm)	20.6 ± 3.9**	18.8 ± 2.7	20.7 ± 3.7	20.6 ± 3.9	20.6 ± 4.3
Ever smoker (former or current), n (%)	14 (23)	0	4 (20)	3 (15)	7 (35)
Systolic blood pressure (mm Hg)	115 ± 26	112 ± 18	117 ± 17	113 ± 32	116 ± 29
Diastolic blood pressure (mm Hg)	73 ± 11*	68 ± 8	74 ± 12	73 ± 10	72 ± 9
Diabetes mellitus, *n* (%)	1 (2)	0	0	1 (5)	0
Raynaud's phenomenon, *n* (%)	16 (27)	9 (15)	4 (20)	5 (25)	7 (35)
Estimated glomerular filtration rate (mL/min/1,73 m^2^)	84 ± 16	Not available	85 ± 14	79 ± 18	87 ± 13
Total cholesterol (mmol/L)	4.7 ± 1.0	4.9 ± 1.1	4.5 ± 1.0	4.7 ± 0.8	4.9 ± 1.1
High-density lipoprotein (HDL) (mmol/L)	1.6 ± 0.5	1.7 ± 0.4	1.5 ± 0.4	1.6 ± 0.5	1.6 ± 0.4
Low-density lipoprotein (LDL) (mmol/L)	2.6 ± 0.8	2.6 ± 0.9	2.5 ± 0.9	2.5 ± 0.7	2.9 ± 0.9
Triglycerides (TG) (mmol/L)	1.1 ± 0.7	1.2 ± 0.6	1.2 ± 0.6	1.3 ± 1.0	0.9 ± 0.4
High-sensitivity CRP (mg/L)	2.2 ± 2.8	2.0 ± 3.7	1.4 ± 1.3	2.7 ± 3.4	2.5 ± 3.2
Anti-dsDNA (IU/mL)	86 ± 200	N/A	71 ± 115	89 ± 200	86 ± 202
Complement protein 3 (g/L)	1.0 ± 0.2	N/A	1.0 ± 0.2	0.9 ± 0.2	1.0 ± 0.2
Complement protein 4 (g/L)	0.2 ± 0.1	N/A	0.1 ± 0.1	0.2 ± 0.1	0.2 ± 0.1
**Medical treatment, ongoing**					
Antimalarial agents, *n* (%)	54 (90)	0	20 (100)	16 (80)	18 (90)
Antihypertensives, n (%)	20 (33)	0	11 (55)	6 (30)	3 (15)
Beta-blockers, *n* (%)	5 (8)	0	1 (5)	1 (5)	3 (15)
ARB/ACE inhibitors, *n* (%)	15 (25)	0	9 (45)	4 (20)	2 (10)
Other antihypertensives, *n* (%)	4 (7)	0	2 (10)	1 (5)	1 (5)
Glucocorticoid therapy *n* (%)	31 (52)	0	12 (20)	9 (45)	10 (50)
*Mean daily Prednisolone dose (mg)*	4.5	0	5.4	3.8	4.2
Warfarin therapy, *n* (%)	11 (18)	0	1 (5)	10 (50)	0
Antiplatelet therapy, *n* (%)	11 (18)	0	5 (25)	6 (30)	0
Statin therapy *n* (%)	5 (8)	0	2 (10)	3 (15)	0
DMARD therapy, *n* (%)	27 (45)	0	11 (55)	9 (45)	7 (35)
Mycophenolate mofetil, *n* (%)	16 (27)	0	11 (55)	4 (20)	1 (5)
Methotrexate, *n* (%)	5 (8)	0	0	1 (5)	4 (20)
Azathioprine, *n* (%)	3 (5)	0	0	2 (10)	1 (5)
Sirolimus, *n* (%)	2 (3)	0	0	1 (5)	1 (5)
Dehydroepiandrosterone, *n* (%)	1 (2)	0	1 (2)	0	0
Biologics, *n* (%)	4 (7)	0	3 (15)	1 (5)	0
Bortezomib, *n* (%)	1 (2)	0	1 (5)	0	0
Rituximab, *n* (%)	1 (2)	0	1 (5)	0	0
Belimumab, *n* (%)	2 (3)	0	1 (5)	1 (5)	0

*Subgroups compared to all controls*.

p < 0.05*, p < 0.01**, p < 0.001***.

*ACE, angiotensin converting enzyme; APS, Antiphospholipid syndrome; ARB, angiotensin II receptor blocker; CRP, C-reactive protein; DMARDs, Disease Modifying Anti-Rheumatic Drugs; LN, lupus nephritis; N/A, not applicable; SDI, SLICC/ACR damage index; SLE, Systemic lupus erythematosus*.

## Results

Basic demographics, laboratory data and ongoing medical therapies are shown in Table 1.

### Intima-Media Thickness

As shown in Table 2, the average IMT (right and left side) of the entire SLE group was increased compared to controls in ICA, CFA, SCA as well as in the aortic arch whereas no significant differences were detected in CCA, SFA and AxA.

**Table 2:** IMT in measured vessels.

	**All SLE (*n* = 60)**	**Controls (*n* = 60)**	**LN (*n* = 20)**	**APS (*n* = 20)**	**Skin and Joint (*n* = 20)**
	**IMT (mm)**	**IMT (mm)**	**IMT (mm)**	**IMT (mm)**	**IMT (mm)**
	**mean ± SD**	**mean ± SD**	**mean ± SD**	**mean ± SD**	**mean ± SD**
**Vessel**					
CCA	0.56 ± 0.10	0.54 ± 0.13	0.54 ± 0.07	0.58 ± 0.11	0.55 ± 0.10
ICA	0.52 ± 0.17^**^	0.45 ± 0.09	0.56 ± 0.20^**^	0.53 ± 0.13^**^	0.47 ± 0.12
SCA	0.58 ± 0.19^*^	0.53 ± 0.13	0.56 ± 0.08	0.61 ± 0.16	0.57 ± 0.13
AxA	0.49 ± 0.10	0.48 ± 0.10	0.50 ± 0.15	0.52 ± 0.15	0.54 ± 0.21
Aortic arch	1.21 ± 0.63^**^	0.98 ± 0.25	1.27 ± 1.05	1.26 ± 0.29^***^	1.11 ± 0.24
CFA	0.57 ± 0.23^**^	0.49 ± 0.11	0.56 ± 0.22	0.62 ± 0.21^**^	0.57 ± 0.22
SFA	0.44 ± 0.11	0.42 ± 0.10	0.44 ± 0.12	0.45 ± 0.10	0.44 ± 0.12

*SLE, systemic lupus erythematosus; APS, antiphospholipid syndrome; IMT, intima media thickness; mm, millimeter; CCA, common carotid artery; ICA, internal carotid artery; SCA, subclavian artery; Axa, axillary artery; CFA, common femoral artery; SFA, superficial femoral artery*.

*Subgroups compared to all controls*.

* *p < 0.05*,

** *p < 0.01*,

*** *p < 0.001*.

In relation to SLE phenotypes, IMT was still increased in the APS group compared to controls in CFA (*p* = 0.006), in the aortic arch (*p* < 0.001) and in ICA (*p* = 0.01). In the LN group, IMT in ICA was increased compared to controls (*p* = 0.002). No significant differences were observed between SLE phenotypes in other vessels.

The reproducibility between two repeated measurements of IMT in the whole group of patients showed a difference of mean 0.06 ± 0.19 mm in right and left CCA (not significant). In the healthy controls this value was 0.08 ± 0.06 (not significant).

### Vessel Wall Assessment

**Figure 2:**
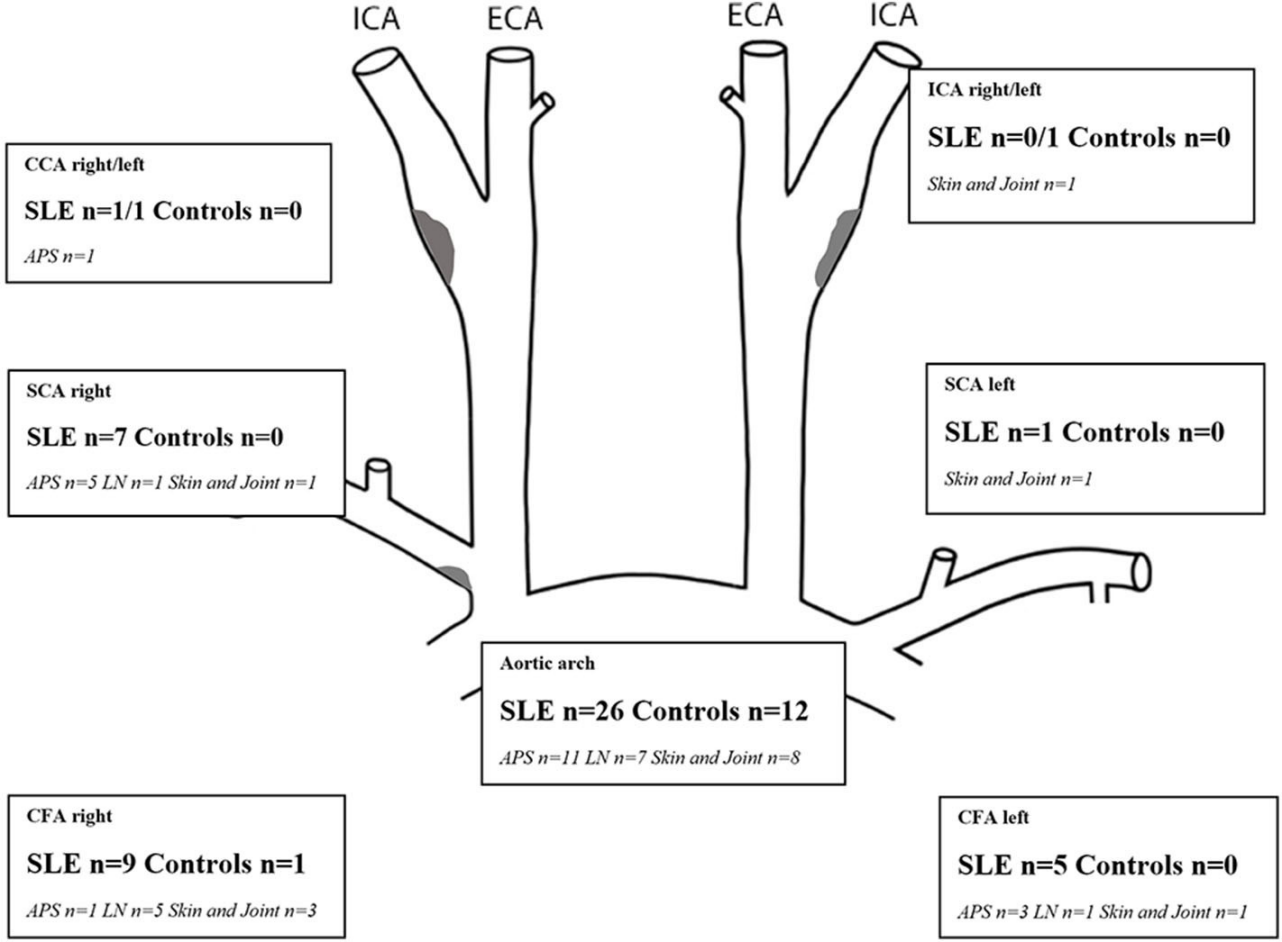
Number of SLE patients with each phenotypic subgroup and controls with areas showing increased IMT (≥0.9 mm for CCA, ICA, SCA, CFA; ≥1.2 mm for the aortic arch). Plaques (gray areas) were distributed as follows: right/left carotid bifurcation: SLE, *n* = 9/5; controls 2/2, proximal SCA right SLE, *n* = 2, CFA right/left SLE, *n* = 3/1. APS, antiphopholipid syndrome; C, controls; CCA, common carotid artery; CFA, common femoral artery; ICA, internal carotid artery; IMT, intima-media thickness; LN, lupus nephritis; mm, millimeter; SCA, subclavian artery; SLE, systemic lupus erythematosus.

Increased IMT ≥0.9 mm observed in SLE patients showed a medium echogenic, homogenous wall thickening that can be seen in inflammatory vascular disease (Figure 1b). This appearance differs from vessel wall changes that can be seen in later stages of atherosclerotic disease, where more irregular wall changes with heterogeneous echogenicity is more common (Figure 1c). The distribution of areas with increased IMT ≥0.9 mm for carotid and central arteries, and IMT ≥1.2 mm in the aortic, is shown in Figure 2. 43% of the SLE cases had an IMT ≥1.2 mm in the aortic arch compared to 20% of the healthy controls (*p* = 0.006). In SCA, CFA, and CCA, increased IMT (≥0.9 mm) was observed in 12% (*p* = 0.002), 15% (*p* < 0.001), and 3% (not significant), respectively in SLE, vs. 0%, 2%, and 0%, respectively among controls. All vessels with increased IMT showed a medium echogenicity without heterogeneous areas. There were no differences regarding vessel wall appearance between patients and controls.

Fibrotic stripes combined with medium echogenic homogenous wall thickening (of inflammatory appearance) (Figure 1d) were only seen in CFA; among 13/60 patients (8%) vs. 3/60 controls (2%) (*p* = 0.007).

**Figure 3:**
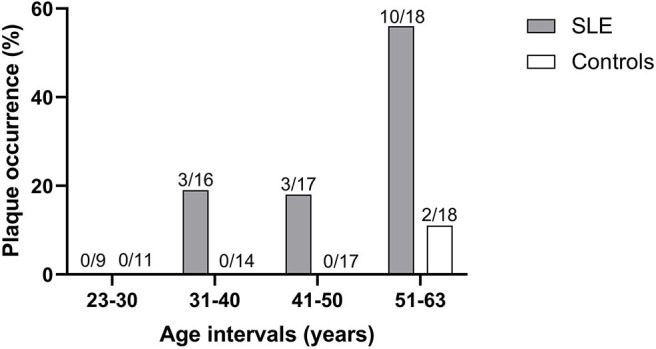
The occurrence of plaques among patients and controls shown in relation to age groups.

**Figure 4:**
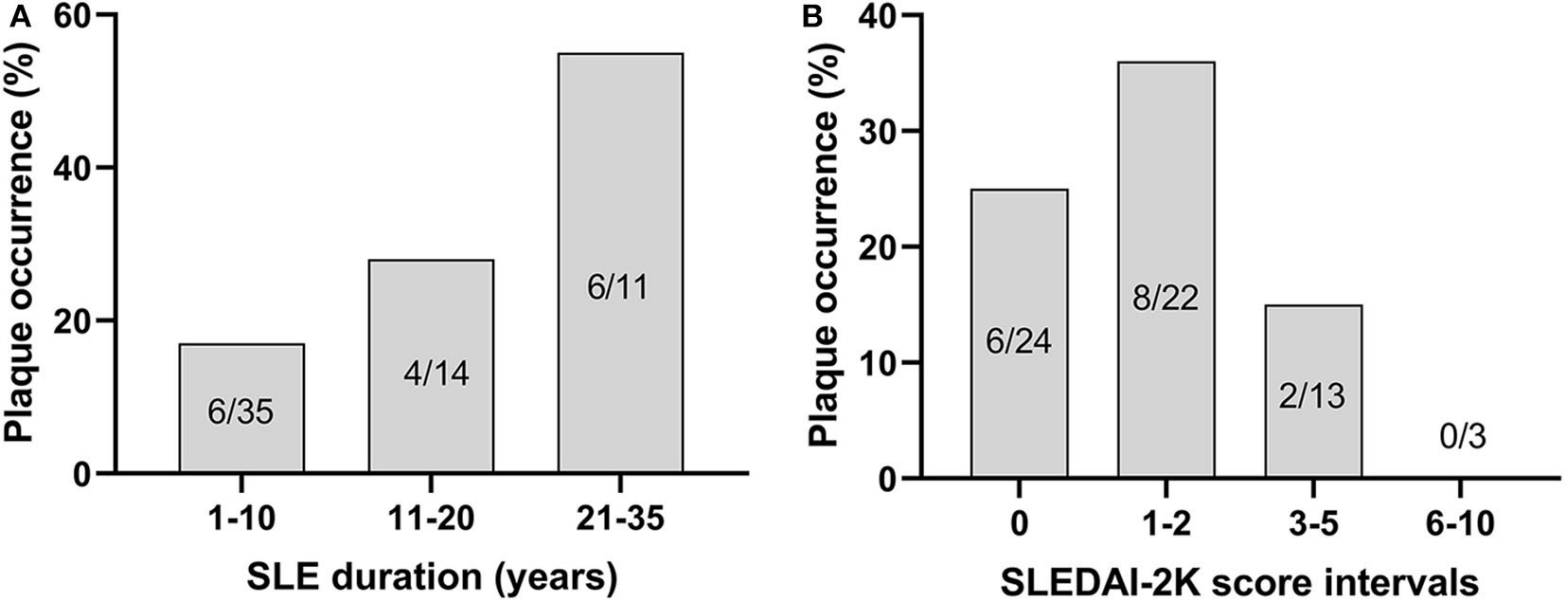
The occurrence of plaques shown in relation to duration of SLE (χ^2^, *p* = 0.05) **(A)** and disease activity, as measured by SLEDAI-2K (not significant) **(B)**.

Fifty percentage of the subjects with echogenic homogenous wall thickening and fibrotic stripes had no atherosclerotic plaques. Atherosclerotic plaques (Figure 1c) were found in 15/60 of SLE cases (25%) and in 2/60 controls (3%) (*p* < 0.001). Plaques were detected among 30% of patients with skin and joint involvement, 25% in LN and 20% in APS. As demonstrated in Figure 3, the plaques occurred at an earlier age among patients compared to controls. The mean age among SLE patients with plaques was 51.4 ± 8.1 years compared to 60.0 ± 2.8 years in controls. The occurrence of plaques was further associated to the duration of SLE (*p* = 0.05), whereas the association with disease activity using SLEDAI-2K was less apparent (illustrated in Figure 4). All plaques had heterogeneous, medium-high echogenicity, which is typical for atherosclerosis. Sixty-seven percentage was located in the carotid bifurcation. Eight percentage of all plaques showed irregular cap. For the CCA and SCA the right side was dominant with plaques in 10 of the patients, and 5 patients had bilateral plaques. In the CFA, plaques were detected in two patients. Two of the healthy controls had bilateral plaques, both in the carotid bifurcation (Figure 2). No significant stenosis was detected, neither among SLE nor in control subjects. Only a few cases of missing data (<0.1%) occurred at the US exams, due to poor visibility or technical problems.

### Relation of IMT to Traditional and SLE Depending Risk Factors

Relation between traditional and disease dependent risk factors and IMT are shown in Table 3. Age was positively related to IMT in all vessels. When all factors in Table 1 (exclusion of diabetes, *n* = 1, and addition of APS and LN) were adjusted for age in a multivariate linear regression model no other factor influenced IMT in the aortic arch or SCA.

**Table 3:** IMT related to background variables, traditional risk factors, laboratory tests A and medical treatment in an univariate regression model for internal carotid artery (ICA), common carotid artery (CCA) and common femoral artery (CFA) among all 60 patients with SLE.

	**All SLE (*****n*** **= 60)**		**All SLE (*****n*** **= 60)**		**All SLE (*****n*** **= 60)**	
	**ICA**		**CCA**		**CFA**	
**Background variables**	**B**	***p*-value**	**B**	***p*-value**	**B**	***p*-value**
Age at examination (years)	0.005	0.003	0.006	<0.001	0.012	<0.001
Female gender	−0.045	0.058	−0.085	0.058	0.074	NS
Duration of SLE (years)	0.005	0.016	0.003	0.053	0.007	0.051
SDI	0.029	NS	0.020	NS	0.065	0.019
**Traditional risk factors and laboratory data**						
Body mass index (BMI) (kg/m^2^)	0.001	NS	0.002	NS	0.022	0.002
Waist circumference (cm)	0.002	NS	0.002	NS	0.010	<0.001
Sagittal abdominal diameter (cm)	0.007	NS	0.003	NS	0.032	<0.001
Ever smoker (former or current)	0.087	0.066	0.012	NS	0.035	NS
Systolic blood pressure (mm Hg)	0.519	NS	0.001	NS	0.002	0.035
Diastolic blood pressure (mm Hg)	0.002	NS	0.002	NS	0.012	<0.001
Raynaud's phenomenon	0.104	0.020	0.095	0.006	0.063	NS
Estimated glomerular filtration rate (mL/min/1,73 m^2^)	−0.03	0.026	0.002	0.082	0.012	<0.001
Total cholesterol (mmol/L)	0.036	0.084	0.043	0.005	0.116	<0.001
High-density lipoprotein (HDL) (mmol/L)	0.019	NS	0.541	NS	0.113	0.078
Low-density lipoprotein (LDL) (mmol/L)	0.028	NS	0.038	0.039	0.087	0.016
Triglycerides (TG) (mmol/L)	0.045	NS	0.057	0.006	0.106	0.008
High-sensitivity CRP (mg/L)	−0.003	0.026	−0.004	0.476	0.022	0.031
**Medical treatment, ongoing**						
Antimalarial agents	−0.105	NS	−0.043	NS	−0.126	NS
Antihypertensives	−0.026	NS	0.003	NS	0.155	0.018
Beta-blockers	0.106	NS	0.079	NS	0.321	0.005
ARB/ACE inhibitors	0.016	NS	0.007	NS	0.170	0.012
Other antihypertensives	−0.083	NS	−0.049	NS	−0.076	NS
Glucocorticoid therapy	−0.018	NS	−0.012	NS	0.092	NS
Mean daily Prednisolone dose (mg)	−0.007	NS	−0.004	NS	0.014	NS
Warfarin therapy	0.003	NS	0.018	NS	0.042	NS
Antiplatelet therapy	−0.010	NS	0.027	NS	0.047	NS
Statin therapy	0.093	NS	0.063	NS	0.213	0.065
DMARD therapy	0.023	NS	0.001	NS	−0.019	NS

*ACE, angiotensin converting enzyme; APS, antiphospholipid syndrome; ARB, angiotensin II receptor- blocker; B, Beta coefficient; CRP, C-reactive protein; DMARD, Disease Modifying Anti-Rheumatic Drugs; SDI, SLICC/ACR damage index*.

In the univariate analysis of CFA, SDI, antihypertensive treatment, β-blocking therapy, Angiotensin II receptor blocker (ARB)/Angiotensin-converting enzyme (ACE) inhibitor treatment, BMI, waist circumference, sagittal abdominal diameter, systolic and diastolic blood pressure, cholesterol, LDL, triglycerides, estimated glomerular filtration rate (GFR) and hsCRP were all related to IMT. However, when all significant variables were included in a multiple linear regression model, age (B = 0.006, *p* = 0.009), sagittal abdominal diameter (B = 0.015, *p* = 0.016), diastolic blood pressure (B = 0.005, *p* = 0.026) and cholesterol (B = 0.052, *p* = 0.031) remained as significant risk factors for IMT in CFA (*R*^2^ = 0.577).

In multiple analysis of CCA, age (B = 0.005, *p* < 0.001), male sex (female B = −0.079, *p* = 0.031) and presence of Raynaud phenomenon (B = 0.066, *p* = 0.026) remained significant (*R*^2^ = 0.395).

In the univariate analysis, duration of SLE significantly influenced IMT in ICA but not in CCA or CFA, where the *p*-values were just above the threshold of significance. However, all significances for disease duration were lost in the multivariate analyses. Smoking habits neither influenced IMT in the univariate nor in the multivariate analyses.

### Relation Between Plaques and Risk Factors

As demonstrated in Table 4, atherosclerotic plaques were significantly and positively related to age, SLE duration, waist circumference, sagittal abdominal diameter, ever smoking, diastolic blood pressure, Raynaud phenomenon, total cholesterol, and triglycerides when patients with plaques were compared to those without.

**Table 4:** Plaque occurrence related to background variables, tradtional risk factors, laboratory variables and ongoing medical treatment analyzed with univariate logistic regression.

	**Patients with plaques**	
	**(*****n*** **= 15)**	
**Background variables**	**B**	***p*-value**
Age at examination (years)	0.106	0.003
Female gender	0.693	NS
Duration of SLE (years)	0.087	0.009
SDI	0.370	NS
**Traditional risk factors and laboratory tests**		
Body mass index (BMI) (kg/m^2^)	0.121	NS
Waist circumference (cm)	0.102	0.001
Sagittal abdominal diameter (cm)	0.227	0.006
Ever smoker (former or current)	2.005	0.003
Systolic blood pressure (mm Hg)	0.011	NS
Diastolic blood pressure (mm Hg)	0.012	<0.001
Raynaud's phenomenon	1.253	0.049
Estimated glomerular filtration rate (mL/min/1,73m^2^)	−0.017	NS
Total cholesterol (mmol/L)	0.721	0.026
High-density lipoprotein (HDL) (mmol/L)	−0.446	NS
Low-density lipoprotein (LDL) (mmol/L)	0.689	0.061
Triglycerides (TG) (mmol/L)	1.115	0.026
High-sensitivity CRP (mg/L)	−0.009	NS
**Medical treatment, ongoing**		
Antimalarial agents	0.560	NS
Antihypertensives	0.847	NS
Beta-blockers	1.682	NS
ARB/ACE inhibitors	0.981	NS
Other antihypertensives	1.196	NS
Glucocorticoid therapy	0.827	NS
Mean daily Prednisolone dose (mg)	0.131	NS
Warfarin therapy	0.145	NS
Antiplatelet therapy	0.189	NS
Statin therapy	0.767	NS
DMARD therapy	0.629	NS

*ACE, angitension converting enzyme; APS, antiphospholipid syndrome; ARB, angiotension II receptor blocker; B, Beta coefficient; CRP, C-reactive protein; DMARD, Disease Modifying Anti-Rheumatic Drugs; SDI, SLICC/ACR damage index*.

When all significant variables were included in a multivariate logistic regression model, age (B = 0.109, *p* = 0.017), waist circumference (B = 0.073, *p* = 0.040) and smoking habits (B = 2.657, *p* = 0.008) remained significant for occurrence of plaques (Nagelkerke *R*^2^ = 0.555).

## Discussion

In this study of well-characterized SLE patients, the great majority with clinically inactive disease, thicker IMT detected with US was observed in ICA, CFA, SCA, and the aortic arch compared to healthy controls, whereas IMT in CCA did not differ. By using this protocol, we were able to detect widespread vascular affection as measured with increased IMT, affected vessel wall appearance, and atherosclerotic plaques. The appearance of the vessel walls in patients with SLE has previously not been studied in detail.

A pathologic cutoff value of IMT ≥0.9 mm was chosen for CCA, ICA, SCA, and CFA according to the latest ESH/ESC hypertension guidelines (29). However, the normal limits of IMT remain a controversial topic. According to the ESH/ESC hypertension guidelines, the relationship of IMT with CVD risk is continuous, and carotid IMT >0.9 mm has been reconfirmed as a marker of asymptomatic organ damage (29). For the aortic arch, a cutoff value of ≥1.2 mm was chosen as IMT in the aortic arch usually is higher than in other vessels based on the findings reported by Bulut et al. albeit this cutoff was based on a group of patients with risk factors for coronary atherosclerosis (30). It is also necessary to keep in mind that IMT is an age-dependent value (31). Normal values in other vessel areas are not well-defined. Concerning CFA, Ayşe et al. studied vessels in patients with CVD risk factors and defined IMT of 1.1 mm as pathological. Furthermore, they showed that IMT in CFA correlated with IMT in CCA (32).

Previous studies used US in SLE for both risk assessment and follow-up (33), but several studies have focused mainly on IMT and the presence of plaques seen in CCA (3, 34, 35). Sporadic studies have evaluated the benefit of US in other vessels (36–38). Herein, we were not able to detect differences in IMT of CCA between patients and controls matched for age and sex, and only 3% of the SLE cases in our study displayed increased IMT ≥0.9 mm in CCA. Other vessels than CCA could be at least as important to investigate.

Areas of increased IMT (≥0.9 mm in CCA, ICA, SCA, and CFA; ≥1.2 mm in the aortic arc) without atherosclerotic plaques showed regular wall thickening of medium echogenicity. We have recently shown that an extended US protocol is of value for the assessment of giant cell arteritis (39) and Takayasu arteritis (28). In these diseases, US appearance of the vessel walls was characterized by a smooth, circumferential, homogenous increased IMT, with or without fibrotic stripes depending on different stages of the disease.

We did not find any differences with regard to vessel wall appearance between SLE patients and matched controls. The intima media among SLE cases was smooth and homogeneous with discrete increased thickness, and fibrotic stripes were usually not observed. This appearance can be seen in inflammatory diseases, with increasing age, as an early sign of atherosclerosis, or due to hypertrophy of the arterial wall (28, 31, 39). The vessel wall changes may thus be discrete but significant in SLE.

The hyperechogenic fibrotic stripes seen in CFA of some patients were similar to those seen in arteritis (28). In arteritis, it is unclear whether these stripes result from early atherosclerosis accompanying inflammation or if they are secondary to the vasculitic process (40). However, the parallel stripes and the smooth homogeneous appearance in vasculitis differ from more irregular findings in atherosclerosis.

The pathogenetic mechanisms of increased IMT in SLE are not clear. Age influenced IMT of all vessel areas. When adjusting for age, only a few traditional and SLE-related risk factors influenced IMT of SCA, AxA, the aortic arch, and SFA. However, in univariate analysis of CFA, IMT was influenced by age, SDI, antihypertensive therapy, β-blocking therapy, ARB/ACE inhibitor treatment, BMI, waist circumference, sagittal abdominal diameter, systolic and diastolic blood pressure, cholesterol, LDL, triglycerides, estimated GFR, and hsCRP. In the multivariate analysis, four factors remained significant as explaining factors for IMT in CFA: age, sagittal abdominal diameter, diastolic blood pressure, and cholesterol. In CCA, age, male sex, and presence of Raynaud remained as significant explaining factors, whereas smoking habits did not. None of the controls and 14/60 SLE cases (23%) had been tobacco smokers, and most of them had finished smoking.

In the univariate analysis only, duration of SLE influenced IMT of ICA but did not reach significance in CCA or CFA (Table 3). Low statistical power and the possibility that disease duration is neutralized by the patients' age are reasonable explanations to why SLE duration did not remain significant in the multivariate analysis. Previous studies indicate that higher age at SLE onset, SLE duration (6, 41), long-time use of corticosteroids, hypercholesterolemia, and postmenopausal status are important with regard to increased CVD risk (6). Long-time use of steroids has been associated with premature atherosclerosis (42). Herein, 52% of patients were prescribed a daily dose of glucocorticoids, but we could not detect any influence on IMT. We were not able to estimate accumulated life-time intake of glucocorticoids, but most patients were in quiescent phase of their disease on a stable dose of steroids during the last 6 months. Ajeganova et al. observed progression of IMT in CCA among patients with SLE during a 7-year surveillance and showed that traditional risk factors, LN, and higher doses of corticosteroids were associated with the progression of IMT (33). By comparing the phenotypic subgroups and controls, we found that IMT of ICA, CFA, and the aortic arch was higher in the APS group. In the LN group, only IMT in ICA was higher compared to controls. However, no significant differences were found between the subgroups.

Concerning occurrence of plaques, similar risk factors were observed as for increased IMT in CFA. Thus, pathogenic mechanisms promoting atherosclerosis seems more likely to contribute to increased IMT than inflammation *per se*. Nevertheless, atherosclerosis and inflammation appear to be closely interrelated (12, 13). Furthermore, several risk factors may influence IMT differently in different arterial areas with varying immunological properties, i.e., divergent Toll-like receptors of dendritic cells are normally distributed in the vessel walls (43).

Measuring IMT with US has low interobserver and intraobserver variability (28, 44), and it has been increasingly used as an indicator of atherosclerosis in clinical and epidemiological research (45). It has been recommended for use as cardiovascular risk assessment by the American College of Cardiology/American Heart association Task force on Practice Guidelines (46). With high-frequency US, it is possible to detect and measure differences of 0.1 mm. In our study, the magnitude of increased IMT in SLE compared to controls was 0.05–0.23 mm in different vessels (Table 2). Differences of IMT in inflammatory diseases, i.e., Takayasu arteritis and giant cell arteritis, may vary several millimeters over time and due to medication. Vessel wall changes in inflammatory disease can be evaluated by different imaging modalities, such as computerized tomography scan, arterial angiography, magnetic resonance imaging (MRI), positron emission tomography (PET), and combined PET-MRI (47). Compared to US, however, these methods have lower resolution, are more expensive, and expose the patient for radiation and have an overall lower availability. For future studies, echocardiography as a complement to the examinations performed in the present study could be considered to achieve an even more complete picture.

The cross-sectional design and the rather low number of examined individuals constitute limitations of the present study. Although the SLE patients were well-matched to the healthy control group, slightly higher measurements regarding waist circumference, sagittal abdominal diameter, and BMI were recorded among the SLE cases. The assessment of the vessel walls and the grading of echogenicity are per definition subjective. Interobserver variability was not possible to study in this investigation as the same sonographer did all online and offline measurements. In contrast, the well-characterized population and the patients' universal access to health care constitute strengths of the present study.

## Conclusion

Among SLE patients without presence of plaques, high-frequency US of multiple arterial areas revealed increased wall thickness with predominantly medium echogenic appearance highlighting possible inflammation or early atherosclerosis. Our findings in CFA emphasize the importance of examining several areas of the arterial tree, which could have implications for clinical practice. Increased number of plaques was observed in SLE compared to age- and sex-matched healthy controls. We found similar risk factors for increased IMT and occurrence of plaques, possibly indicating atherosclerotic mechanisms rather than inflammation, but atherosclerosis and inflammation appear to be closely interrelated. These data call for confirmation, and careful follow-up is needed before firm conclusions regarding cardiovascular management can be drawn.

## Data Availability Statement

All datasets generated for this study are included in the article/Supplementary Material.

## Ethics Statement

The studies involving human participants were reviewed and approved by The Regional Ethics board in Linköping. The patients/participants provided their written informed consent to participate in this study.

## Author Contributions

CSv: study design, methodology, investigation, formal analysis, and manuscript writing. PE and CSj: study design, investigation, formal analysis, manuscript writing, and supervision. HZ: study design, methodology, investigation, formal analysis, manuscript writing, and supervision. All authors contributed to the article and approved the submitted version.

## Conflict of Interest

The authors declare that the research was conducted in the absence of any commercial or financial relationships that could be construed as a potential conflict of interest.

## Abbreviations

ACE: Angiotensin-converting enzyme
APS: Antiphospholipid syndrome
ARB: Angiotensin II receptor blocker
aPL: Antiphospholipid antibodies
AXA: Axillar artery
CCA: Common carotid artery
CFA: Common femoral artery
hsCRP: High sensitive C-reactive protein
CVD: Cardiovascular disease
HDL: High-density lipoprotein
ICA: Internal carotid artery
IMT: Intima-media thickness
KLURING: Clinical Lupus Register In North-eastern Gothia
LDL: Low-density lipoprotein
LN: Lupus Nephritis
MRI: Magnetic resonance imaging
PET-MRI: Positron emission tomography
SCA: Subclavian artery
SFA: Superficial femoral artery
SLICC: Systemic Lupus International Collaborating Clinics
SLE: Systemic lupus erythemathosus
US: Ultrasound.
