# Supplementary material for: High-Frequency Ultrasound of Multiple Arterial Areas Reveals Increased Intima Media Thickness, Vessel Wall Appearance, and Atherosclerotic Plaques in Systemic Lupus Erythematosus
Source: Front Med (Lausanne). 2020 Oct 9;7:581336. doi: 10.3389/fmed.2020.581336 (PMC7581856; doi:10.3389/fmed.2020.581336)
Supplement: Supplementary file 1 [file Table_1.docx]

**Supplementary Table 1.** Frequencies of fulfilled 1982 ACR criteria among the 60 included patients with SLE.

|  | **Frequency (%)** |
| --- | --- |
| **1. Malar rash** | 24 (40) |
| **2. Discoid rash** | 3 (5) |
| **3. Photosensitivity** | 27 (45) |
| **4. Oral ulcer** | 13 (22) |
| **5. Arthritis** | 47 (78) |
| **6. Serositis** | 23 (38) |
| *a) Pleuritis* | 21 (35) |
| *b) Pericarditis* | 19 (32) |
| **7. Renal disorder** | 20 (33) |
| *a) Proteinuria* | 20 (33) |
| *b) Cellular casts* | 18 (30) |
| **8. Neurological disorder** | 5 (8) |
| *a) Seizures* | 5 (8) |
| *b) Psychosis* | 1 (2) |
| **9. Hematologic disorder** | 40 (67) |
| *a) Hemolytic anemia* | 3 (5) |
| *b) Leukopenia* | 20 (33) |
| *c) Lymphopenia* | 29 (48) |
| *d) Thrombocytopenia* | 6 (10) |
| **10. Immunologic disorder** | 39 (65) |
| *a) Anti-dsDNA* | 34 (57) |
| *b) Anti-Sm* | 6 (10) |
| **11. Antinuclear antibodies*** | 60 (100) |
| **Number of fulfilled ACR criteria**  (median, range) | 5 (3–9) |

* Positive by immunofluorescence microscopy (IF-ANA)
